# Alcohol use disorder severity and reported reasons not to seek treatment: a cross-sectional study in European primary care practices

**DOI:** 10.1186/s13011-015-0028-z

**Published:** 2015-08-12

**Authors:** Charlotte Probst, Jakob Manthey, Alicia Martinez, Jürgen Rehm

**Affiliations:** Social and Epidemiological Research Department, Centre for Addiction and Mental Health, 33 Russell Street, Toronto, ON M5S 2S1 Canada; Institute for Clinical Psychology and Psychotherapy, Technische Universität Dresden, Chemnitzer Str. 46, 01187 Dresden, Germany; Department of Addiction, Hospital Clínic, University of Barcelona, Carrer Villarroel 170, 08036 Barcelona, Spain; Addiction Policy, Dalla Lana School of Public Health, University of Toronto, 155 College Street, Toronto, ON M5T 3M7 Canada; Institute of Medical Science, University of Toronto, 1 King’s College Circle, Toronto, ON ON M5S 1A8 Canada; Department of Psychiatry, University of Toronto, 250 College Street, Toronto, ON M5T 1R8 Canada

**Keywords:** Alcohol use disorder, Alcohol use disorder severity, Treatment seeking, Treatment barrier, Stigmatization, Primary health care, Europe, Health services needs and demand

## Abstract

**Background:**

Alcohol use disorders are among the mental disorders with the lowest treatment rates. Increasing the treatment rates requires insight on the reasons why patients do not seek treatment. This study examined self-reported reasons for not seeking treatment and their association with alcohol use disorder severity among primary health care patients diagnosed with an alcohol use disorder.

**Methods:**

Alcohol use disorders, health service utilization, and reasons for not seeking treatment were assessed via interviews on regionally representative samples of primary care patients from 6 European countries (Italy, Germany, Hungary, Latvia, Poland and Spain, total *N* = 9,098). Additionally, general practitioners had to fill in a questionnaire assessing their patients’ alcohol use and alcohol use disorders. A multinomial logistic regression was performed to investigate the association between reasons for not seeking treatment and alcohol use disorder severity.

**Results:**

Of 1,008 patients diagnosed with an alcohol use disorder (via general practitioner or patient interview) in the past 12 months, the majority (*N* = 810) did not receive treatment and 251 of those gave a reason for not seeking treatment. The most frequent reason was ‘lack of problem awareness’ (55.3 % of those who responded), the second most common response was ‘stigma or shame’ (28.6 %), followed by ‘encounter barriers’ (22.8 %) and ‘cope alone’ (20.9 %). The results indicated lower probabilities of reporting ‘denial’ and higher probabilities to report ‘encounter barriers’ as alcohol use disorders severity increases. However, both trends were discontinued for patients with severe alcohol use disorders.

**Conclusions:**

Particularly at lower levels of alcohol use disorder severity, a lack of problem awareness prevents patients from seeking treatment. Routinely alcohol consumption monitoring in primary care practices could help primary and secondary prevention of alcohol use disorders and increase treatment coverage.

**Electronic supplementary material:**

The online version of this article (doi:10.1186/s13011-015-0028-z) contains supplementary material, which is available to authorized users.

## Background

Alcohol use disorders (AUDs) are among the mental disorders with the lowest treatment rate. In high income countries 10 % or less of the people fulfilling the diagnostic criteria receive treatment [1] (Europe), [2] (Europe), [3] (USA). Given that alcohol is one of the most important risk factors for morbidity and mortality in Europe [4] and AUDs account for significant shares of disease burden in Europe [5], increasing treatment engagement should be a public health priority [6]. Rehm and colleagues recently demonstrated that increasing the treatment coverage to 40 % would lead to a reduction of up to 10 % in alcohol-attributable mortality in the EU within the first year alone [5]. One way to increase treatment rates is to increase patients’ treatment seeking. It is therefore crucial to know why patients do not seek treatment. The present study examined patients’ self-reported reasons for not seeking treatment and their association with AUDs severity. Past research concentrated on three explanations [7]:

- Patients fear stigmatization
- Patients do not believe treatment is effective/helpful or do not know about treatment options
- Patients deny having a problem with their alcohol use or want to cope with it on their own.

As postulated by the modified labelling theory, people attempt to avoid a stigma (e.g. via closure or social withdrawal) [8]. In many modern societies, people labelled as ‘alcoholics’ are regarded as being responsible for their situation, unpredictable and dangerous [9]. Research has repeatedly shown that people with AUDs experience stigmatization (by the public as well as from health professionals) more severely than people with other mental disorders [10, 9]. A high perceived stigma in persons diagnosed with an AUD has been shown to reduce the probability of using health care services [11–13] and thereby contributes to a decreased likelihood of treatment seeking.

Another reason for not seeking treatment is denial or lack of problem awareness. Several studies showed that patients did not seek treatment because they did not perceive any need for treatment [14–16, 3]. Even if acknowledged, many persons with AUDs expressed the wish to handle the problem on their own instead of seeking professional treatment [17, 3].

Skepticism about the treatments system has also been shown to prevent seeking treatment: patients reported doubts about the effectiveness of AUDs treatment and expressed the wish not to stop drinking completely [13]. Actual treatment-related barriers like financial barriers (e.g. affordability, insurance issues) or structural barriers (e.g. lack of knowledge who to ask for help, lack of time) have also been reported repeatedly by patients [12, 18–20, 3].

**Fig. 1:**
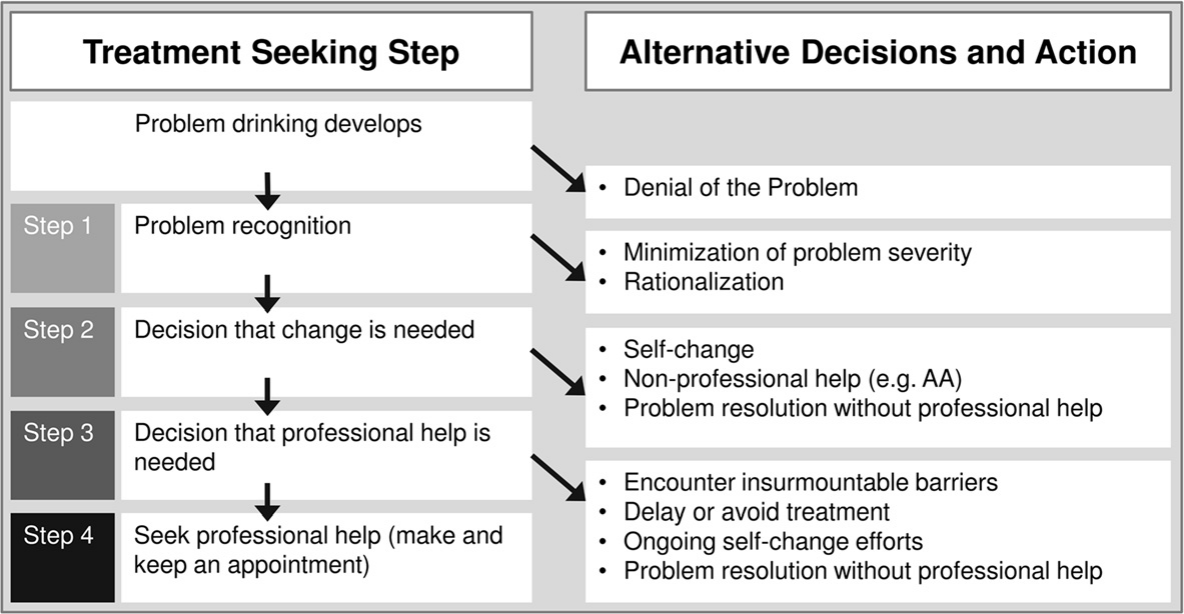
Theoretical framework of the treatment seeking process as suggested by (Saunders et al. [17]). Each step in the process of seeking treatment for an alcohol use disorder is related to specific reasons for not seeking treatment. Reprinted from Journal of Substance Abuse Treatment, 30 (3), Saunders, Zygowicz, & D'Angelo, Person-related and treatment-related barriers to alcohol treatment, p. 261–270, © 2006, with permission from ELSEVIER

It should be noted that some of the reasons mentioned above presuppose a certain degree of problem insight while others do not. In a theoretical framework (see Fig. 1), Saunders linked the treatment seeking process and related barriers to the degree of problem insight [21, 22, 17]. According to this framework, at an early stage of AUDs people deny having a problem with alcohol or do not recognize their drinking behavior as problematic. However, once the problem is acknowledged, those affected might wish to handle it on their own, partly because of their fear of stigmatization. Previous research has shown that patients that seek treatment do so after postponing treatment seeking for several years and a number of failed attempts to quit or control drinking on their own [23]. Those with more severe symptoms might encounter barriers like accessibility/affordability once they recognize that treatment seeking is necessary.

Building on this framework, we do emphasize the relationship between the reasons for not seeking treatment and AUDs severity [17, 24, 25]. We hypothesized that at low levels of AUDs severity a lack of problem awareness is the predominant reason not to seek treatment. As AUDs severity increases, denial becomes more difficult and individuals are more likely to consider treatment and to face actual barriers associated with the treatment seeking process.

It should be noted that while evidence for Europe is sparse, the large part of the evidence on treatment seeking is based on studies from North American countries (particularly based on the two large epidemiological studies National Epidemiologic Survey on Alcohol and Related Conditions (NESARC) [3], and the National Household Survey on Drug Use and Health (NSDUH) [26] from the US). The present study is thereby a necessary addition of evidence for European countries.

## Methods

The Alcohol Dependence in Primary Care study (APC study) collected data between January 2013 and January 2014. General practitioners (GPs) were asked to evaluate their patients’ alcohol use as well as other health related variables and patients were interviewed independently by trained study personnel. While the most important features are stated below, more details on design, sampling, and assessment are described elsewhere [27–29].

### Sampling

GPs and their patients were representatively sampled in nine regions from six European countries (Italy, Germany, Hungary, Latvia, Poland and Spain) using complete registers or local GP associations. In some countries, GPs were randomly sampled from registers while in others sampling was stratified according to pre-selected criteria (e.g. urbanity, size of GP practice). 56.4 % of contacted GPs refused study participation. The majority of GPs refused their participation for reasons unrelated to this study’s characteristics, e.g. tight schedules, insufficient space in the PC facility, lack of interest in research activities generally or current participation in other studies. For countries with populations below 40 million inhabitants (Latvia and Hungary) primary care practices were sampled to be nationally representative. In the remaining countries primary care practices were regionally representative (Italy: Friuli-Venezia Giulia and Tuscany region; Germany: Saxony and Berlin state; Poland: Łódź and Podkarpackie province; Spain: Catalonia autonomous community). In Hungary and Spain, all patients with GP assessment were interviewed. In the remaining countries, patients were selected for interview either randomly or based on stratification (oversampling AUDs cases as judged by the GP), with an overall patient refusal rate of 17.8 %. Ethical approval was obtained in each of the participating countries and the respective reference numbers are shown in Additional file 1: Table S1 as Additional file.

### Assessment

GPs were asked to fill in a brief questionnaire for all patients (18–64 years of age) coming to their practice on a given preselected day or several days. Information on the patients’ demographics, health status, reasons for consulting the GP, current alcohol use and AUDs, and information regarding treatment for AUDs were determined by the GP, based on his/her best knowledge of the patients’ problems. GPs were allowed to use the patients’ medical charts. Trained study personnel interviewed the patients after their appointment at the GP’s premises or on a subsequent day at the study center. Informed consent was obtained by all interviewed patients.

The patient interview included the AUDs section of the Composite International Diagnostic Interview (CIDI); [30] assessing information on alcohol use and AUDs criteria according to the fourth as well as the fifth revision of the Diagnostic and Statistical Manual of Mental Disorders (DSM-IV; [31], DSM-5); [32]. To assess reasons for not seeking treatment, patients reporting any DSM-5 AUDs symptom, were subsequently asked if they sought help. If they responded negatively they were asked an open ended (‘If you did not ask for help, please describe why’; lifetime and 12 months) as well as a set of closed questions (asking for shame, stigma, dissatisfaction with treatment system and wish not to stop drinking completely; referring to the past 12 months only). Additionally, the patient interview comprised questions on socio-demographics as well as a number of validated questionnaires.

### Coding procedure

A coding scheme was developed in a directed content analysis [33]: First a literature review identifying key reasons for not seeking treatment was performed to generate the basic coding scheme. Then all answers on the open ended question were read to identify meaningful subcategories and include reasons not yet covered by the basic coding scheme. After coding a sample of 100 answers the authors discussed and adapted the scheme where necessary to capture all reported reasons. The coding scheme (see Additional file 1: Table S2) was then applied independently by two raters. All cases of non-agreement were revisited and discussed between the authors to come to a final decision.

The scheme allowed for multiple coding on the following broader categories (number of subcategories): ‘lack of problem awareness’ (6), ‘cope alone’ (4), ‘stigma or shame’ (3), ‘other prominent problem’ (2), ‘encounter barriers’ (7), and ‘not codable’ (2). Answers were classified as ‘not codable’ when the patient did report seeking professional help or when the answer was incomplete or incomprehensible.

Comprehensive variables were then generated including the information on treatment seeking assessed in closed variables as well as the coded answers from open ended questions (see Additional file 1: Table S2). For the evaluation of Saunders’ theoretical framework [17] we summarized the reported reasons in the following four categories: (1) ‘denial of the problem’; (2) ‘minimization of problem severity’; (3) ‘self-change’; and (4) ‘encounter barriers’. See Table 1 for an overview on broader categories, subcategories and classification according to Saunders’ framework.

**Table 1:** Categorization of reasons for not seeking treatment and matching of reasons reported in the present study to Saunders' theoretical framework

Saunders’ framework	Present study	
Reasons related to the four steps of treatment seeking	Broader category	Subcategories
Denial of the problem	Lack of problem awareness	Drinking was no problem
		No help needed
		Normal consumption
		Consumption under control
		Nothing serious, no problem insight
Minimization of problem severity, rationalization	Other problem	Other problem was more prominent
		Other substance problem was more prominent
	Cope alone	Acknowledgement of problem but not of its severity
Self-change, problem resolution without professional help, non-professional help	Cope alone	Wish to cope alone
		Reduction in drinking
		Non-professional support
Encounter insurmountable barriers; delay or avoid treatment, ongoing self-change efforts	Stigma or shame	Fear of stigma and shame
		Social pressure
		Drinking is private issue
	Encounter barriers	Lack of possibility or knowledge
		Lack of time
		Asked for help but did not get any
		Treatment was not affordable
		Wish not to stop drinking
		Treatment was not seen as an option
		No trust in treatment system
		The help needed was not offered

### Statistical methods

Inter-rater agreement was evaluated using kappa statistics. Kappa was calculated for accordance on the broader category (e.g. any kind of lack of problem awareness was coded vs. no lack of problem awareness coded) as well as accordance on subcategories within broader categories [34].

Frequencies and percentages were calculated for all patients who were diagnosed with an AUD by their GP or by CIDI (at least a mild AUD as per DSM-5), had not received treatment, and had a valid answer in the questions concerning treatment seeking. Exploratory logistic regressions were calculated to investigate patient characteristics (age, sex, years of education, employment status, and AUDs severity) predictive of providing a valid answer in the questions concerning treatment seeking. Percentages and frequencies of broader categories by country were calculated for the past 12 months. To investigate country specific outliers, standardized residuals [35] were calculated. A standardized residual > |3| was considered as outlier [35]. The country specific results are reported in the Additional file 1: Table S3 and should be interpreted with caution, because of the small sample size.

To investigate associations with AUDs severity, the reason for not seeking treatment ordered according to Saunders’ framework (see above) served as dependent variable. AUDs severity was operationalized as defined in DSM-5 (sub threshold (1 criterion), mild (2–3 criteria), moderate (4–5 criteria), severe (6 or more criteria)). As the proportional odds assumption did not hold, multinomial logistic regression was performed introducing AUDs severity as a factorial variable and adjusting for age. Based on the resulting model, predicted probabilities were calculated for all levels of AUDs severity. All analyses were conducted in STATA 12, considering 12 months and lifetime (excluding 12 months) variables separately. Sample weights were applied in all analyses to account for different sampling probabilities [27, 28].

## Results

### Inter-rater accordance

The inter-rater accordance was assessed based on all coded answers (*N* = 552 for past 12 months questions and *N* = 1,107 for lifetime), irrespective of the respondent’s treatment status and AUDs diagnosis (i.e. sub threshold cases with only one criterion included). Multiple answers per patient were possible. The two raters showed a substantial to perfect accordance as shown in Table 2.

**Table 2:** Inter-rater accordance (Kappa) between two independent raters on all answers reported. Multiple answers per patient were possible across broader categories but not within each category

	Broader category		Within category	
	12 months (*N* = 552)	Lifetime (*N* = 1107)	12 months (*N* = 552)	Lifetime (*N* = 1107)
Category of reason for not seeking treatment	Kappa	Kappa	Kappa	Kappa
Lack of problem awareness	0.89	0.86	0.87	0.88
Cope alone	0.82	0.83	0.77	0.81
Stigma or shame	0.89	0.79	0.89	0.79
Other prominent problem	1.00	0.86	1.00	0.84
Encounter barriers	0.89	0.80	0.87	0.60

all Kappas have a p-value < 0.001

### Frequencies and percentages

The majority of the 9,098 patients (3,715 males and 5,383 females) interviewed came from Hungary (*N* = 2,306), followed by Spain (*N* = 1,994). The remaining patients came from Germany (*N* = 1,356), Latvia (*N* = 1,302), Poland (*N* = 1,197) and Italy (*N* = 943). The mean age was 44.3 years (standard deviation 13.3 years), ranging between 18 and 64 years. On average the patients received 12.7 years of education (standard deviation 3.6 years) and 13.3 % of the sample was unemployed. Details on patients demographics are reported elsewhere [27].

**Fig. 2:**
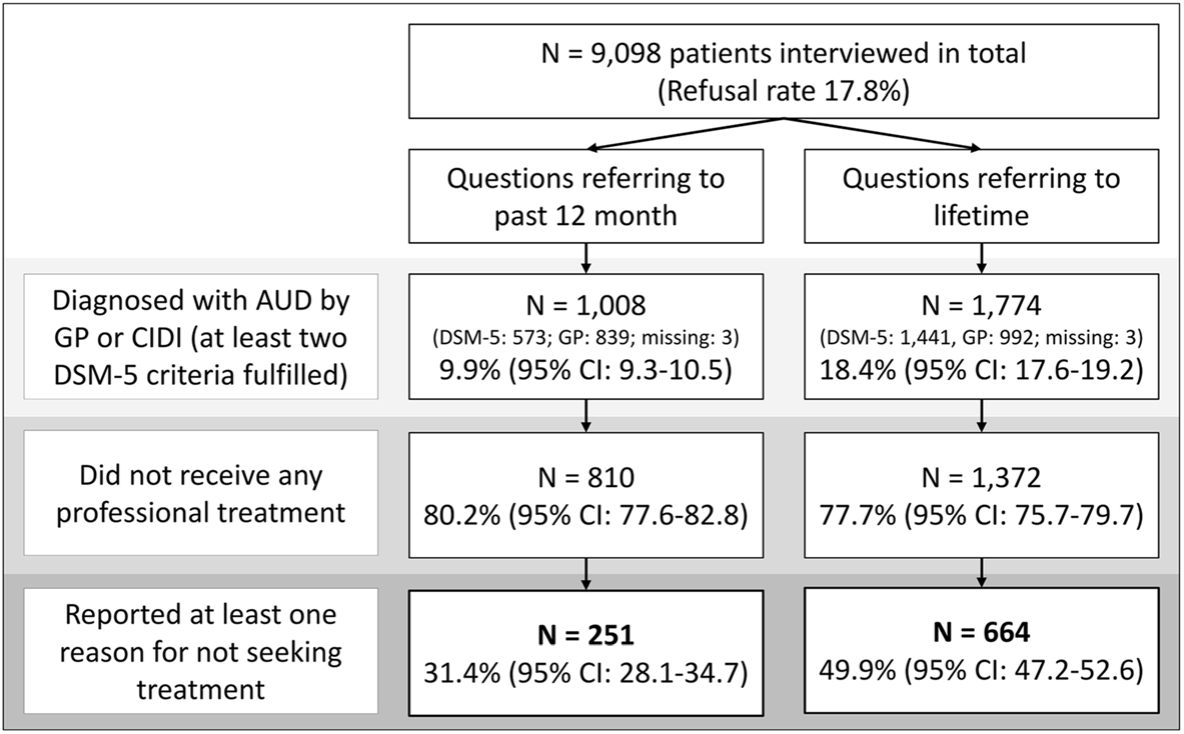
Flow chart for frequencies and weighted percentages of patients enclosed in the main analysis. 95 % confidence intervals (CI) are indicated in brackets. Patients diagnosed with an alcohol use disorder (AUD) by their general practitioner (GP) or by the Composite International Diagnostic Interview (CIDI) (at least 2 DSM-5 criteria fulfilled), who did not receive treatment, and reported at least one reason for not seeking treatment were included

As shown in Fig. 2, 1,008 patients were diagnosed with an AUD (12 months). 80 % of those patients did not receive any professional treatment [28] and of those 251 reported a reason why they did not receive treatment. Of the 1,774 patients with a lifetime AUD diagnosis (12 months excluded), 77 % did not receive treatment and 664 reported a reason for not seeking treatment. For questions referring to the past 12 months AUDs, unemployed patients were more likely to report a reason for not seeking treatment (OR = 2.24, 95 % confidence interval (CI) 1.36-3.70) and patients with a severe AUD were less likely to report a reason for not seeking treatment (OR = 0.53, 95 % CI 0.31-0.92). For lifetime AUDs no patient characteristic was predictive of reporting a reason for not seeking treatment.

Frequencies (multiple answers per patient possible) and weighted percentages including CIs of categories and subcategories are shown in Table 3. For past 12 months cases, the most frequent reason for not seeking treatment was ‘lack of problem awareness’ (55.3 %). Patients reporting ‘lack of problem awareness’ most frequently, did not consider their drinking as a problem or stated that no help was needed. The second most common reasons was ‘stigma or shame’ (28.6 %), closely followed by ‘encounter barriers’ (22.8 %) and ‘cope alone’ (20.9 %). The specific barrier reported most frequently was the wish not to stop drinking (72.9 %). Among patients fulfilling at least four DSM-5 criteria (moderate to severe AUDs), the share of patients reporting ‘lack of problem awareness’ was 52.1 % (12 months, 95 % CI 39.1-65.1; not shown in Table 3).

**Table 3:** Frequencies and weighted percentages including 95 % confidence intervals (CI) for reasons not to seek treatment. Refers to patients that were diagnosed with an alcohol use disorder, reported a reason for not seeking treatment, and that did not receive treatment. Multiple reasons per patient were possible. Percentages in subcategories refer to respective broader category

	12 months			Lifetime (excluding past 12 months)		
Reason for not seeking treatment	N	%	95 % CI	N	%	95 % CI
Lack of problem awareness	139	55.3	49.0 - 61.2	519	78.3	75.1 - 81.4
1 Drinking was no problem	50	37.4	29.1 - 45.8	221	42.5	38.2 - 46.8
2 No help needed	59	41.3	32.3 - 49.8	168	32.4	28.3 - 36.5
3 Normal consumption	9	6.4	2.2 - 10.5	47	9.3	6.7 - 11.9
4 Consumption under control	11	8.3	3.5 - 13.2	55	10.8	8.1 - 13.5
5 Nothing serious, no problem insight	10	6.5	2.4 - 10.6	28	5.0	3.2 - 6.9
Cope alone	53	20.9	15.8 - 26.1	115	17.2	14.8 - 20.1
1 Wish to cope alone	47	86.7	76.3 - 97.1	77	69.4	66.7 - 75.5
2 Reduction in drinking	2	5.1	0.0 - 12.3	9	8.0	2.9 - 13.1
3 Non - professional support	0	0.0	-^a^	9	8.0	2.9 - 13.1
4 Acknowledgement of problem but not of its severity	4	8.2	0.0 - 16.4	20	17.3	10.2 - 24.3
Stigma or shame	73	28.6	22.9 - 34.4	16	2.4	1.2 - 3.6
1 Fear of stigma and shame	71	97.2	92.3 – 100	10	62.1	35.2 - 89.0
2 Social pressure	0	0.0	-^a^	4	25.1	1.1 - 49.1
3 Drinking is private issue	2	2.8	0.0 - 6.7	2	12.8	0.0 - 31.3
Other prominent problem	3	1.3	0.0 - 2.8	11	1.7	0.7 - 2.7
1 Other problem was more prominent	2	68.3	0.0 - 100	6	53.4	18.1 - 88.7
2 Other substance was more prominent	1	31.7	0.0 - 100	5	46.6	11.3 - 81.9
Encounter barriers	60	22.8	17.6 - 28.1	31	4.6	3.0 - 6.3
1 Lack of possibility or knowledge	2	3.0	0.0 - 7.3	11	35.8	17.7 - 54.0
2 Lack of time	0	0.0	-^a^	2	6.6	0.0 - 16.1
3 Asked for help but did not get any	0	0.0	-^a^	1	3.3	0.0 - 10.1
4 Treatment was not affordable	3	4.7	0.0 - 10.1	1	4.1	0.0 - 12.3
5 Wish not to stop drinking	43	72.9	61.2 - 84.6	3	10.0	0.0 - 21.3
6 Treatment was not seen as an option	3	4.0	0.0 - 8.8	7	20.2	5.5 - 34.9
7 No trust in treatment system	6	9.9	2.1 - 17.6	6	20.0	4.8 - 35.2
8 The help needed was not offered	3	5.6	0.0 - 12.0	0^b^	0.0	-^a^

^a^The confidence interval was not calculable

^b^Barrier 8 (‘The help needed was not offered’) was assessed in a closed question for the past 12 months only

Percentages of broader categories do not add up to 100 % because multiple answers were possible

Country specific analyses showed a higher than expected prevalence for Germany (‘lack of problem awareness’), Latvia (‘cope alone’), and Spain (‘shame or stigma’ and ‘encounter barriers’) as indicated by standardized residuals above three. Lower than expected prevalence was only observed in Spain (‘lack of problem awareness’). Country specific percentages and standardized residuals can be found in Additional file 1: Table S3.

For lifetime AUDs 78.3 % of the patients named ‘lack of problem awareness’ as a reason for not seeking treatment. The second most common answer was ‘cope alone’ (17.2 %), while the remaining categories were all below 5 %.

### The association with alcohol use disorder severity

**Fig. 3:**
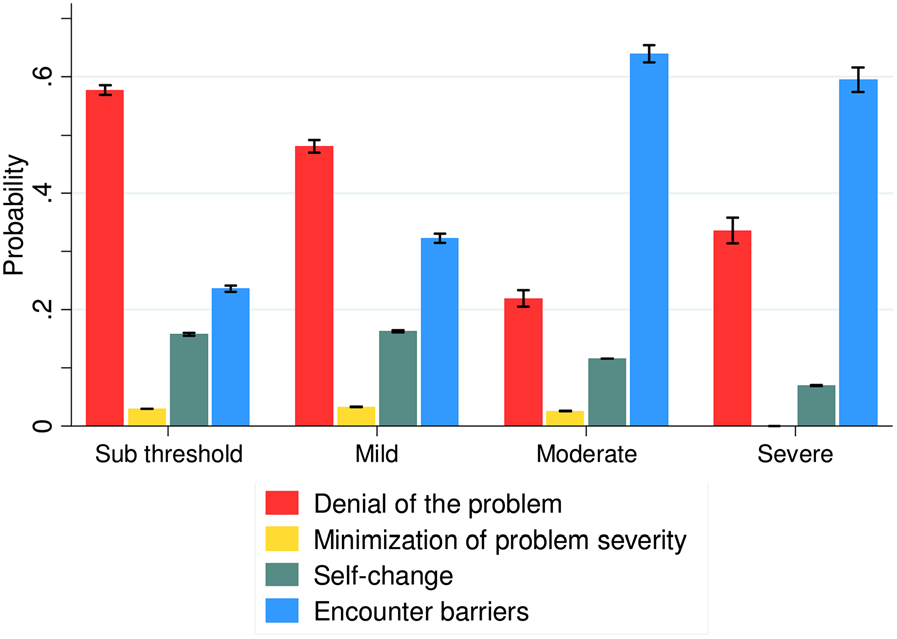
Probabilities of reporting reasons for not seeking treatment, predicted by alcohol use disorder severity. Severity of alcohol use disorders in the past 12 months ranged from sub threshold (reference) to severe as defined by DSM-5. Predictions are based on multinomial logistic regression. Reasons are grouped by Saunders' theoretical framework. 95 % confidence intervals are indicated

Multinomial logistic regression was conducted to test the hypothesis that severity of AUDs (12 months) was associated with the reported reason for not seeking treatment, categorized according to Saunders’ theoretical framework (1= ‘denial of the problem’, 2 = ‘minimization of problem severity’, 3 = ‘self-change’, 4 = ‘encounter barriers’; see Table 1). The analysis included sub threshold cases fulfilling only one DSM-5 criterion and therefore included more patients than the descriptive analyses reported above. Overall 379 patients were included in the analysis of which 192 had a sub threshold, 140 a mild, 40 a moderate and 25 a severe AUD. The results are shown in Table 4. Based on the model probabilities for reporting a certain reason for not seeking treatment were calculated for each level of severity as defined by DSM-5. The results shown in Fig. 3 indicate lower probabilities of reporting ‘denial’ and higher probabilities to report ‘encounter barriers’ as AUDs severity increases. However, both trends were discontinued for patients with severe AUDs.

**Table 4:** Results from multinomial logistic regression. Reported reason not to seek treatment was predicted by alcohol use disorders (AUDs) severity (past 12 months), adjusting for age. Odds ratios (OR), respective 95 % confidence intervals (CI) and p-values are displayed

	Reason for not seeking treatment					
	Minimization of problem severity vs. denial		Self-change vs. denial		Encounter barriers vs. denial	
AUDs^a^ severity	OR (95 % CI)	p-value	OR (95 % CI)	p-value	OR (95 % CI)	p-value
Mild vs. sub threshold	1.35 (0.33 - 5.46)	0.674	1.26 (0.67 - 2.36)	0.471	1.67 (0.99 - 2.83)	0.097
Moderate vs. sub threshold	2.39 (0.25 - 23.00)	0.448	2.04 (0.57 - 7.31)	0.272	7.70 (3.17 - 18.71)	<0.001
Severe vs. sub threshold	n.a.^b^	n.a.^b^	0.78 (0.16 - 3.97)	0.773	4.58 (1.82 - 11.56)	0.001

^a^Alcohol use disorder severity as defined by the fifth revision of the Diagnostic Statistical Manual

^b^Not applicable (no observations for minimization of problem severity)

As also reflected in the odds ratios from the multinomial regression, the predicted probabilities of reporting ‘minimization of problem severity’ or ‘self-change’ were overall relatively low and did not show a clear association with AUDs severity.

## Discussion

### Interpretation

The present study investigated reasons for not seeking treatment on a sample of European primary care patients that were diagnosed with an AUD but did not receive any professional AUDs treatment. We were able to show that the reasons for not seeking treatment were related to AUDs severity. However, half of the patients that fulfilled at least four DSM-5 AUDs criteria in the past 12 months still stated that they did not have a problem with alcohol use requiring treatment. The finding that the main reason for not seeking treatment was a lack of acknowledging the problem was in line with studies from the US [26]. As discussed below, the results indicate a huge potential for prevention when the health care system would address those patients with mild AUDs that are already at increased risk but do not (yet) perceive a need to seek treatment [36].

### Implications

The present study showed once more that patients are unlikely to recognize and acknowledge problems related to their alcohol use themselves. Thereby a continuous monitoring of patients’ alcohol use could be useful not only for prevention of a number of physical health consequences but also for the primary as well as secondary prevention of AUDs. A Swedish study investigated frequencies of life style advice in GP settings in a large sample of primary care patients [37]. Across all age groups patients were least likely (only 4.7 % of the patients) to receive advice about alcohol compared to other life style areas like diet, smoking, or exercise.

The screening and monitoring of patients’ blood pressure could serve as a model for routine alcohol use assessment in primary care [38]. The latter practice has furthermore been argued to decrease stigmatization of AUDs [39]: instead of classifying patients into two distinct categories of ‘healthy and diseased’ each patients’ alcohol use behavior would be allocated on a continuum of related risk.

As reported in more detail in other publications [27, 28] the present study showed that GPs were more likely to detect AUDs when they became evident in decreased liver functioning and other physical indicators. Again, with a routine assessment of patients alcohol use, GPs might be able to offer brief interventions before more severe consequences emerge. Brief interventions (in primary care) have been shown to be highly effective and useful in prevention of progression into more severe stages of AUDs, alcohol-related mortality and societal costs [40–43].

The present study also showed that for more severe cases of AUDs, treatment barriers were reported by the majority of the patients as reasons not to seek treatment. This indicates that even once patients acknowledged a need for treatment they might face barriers as a lack of information or high costs. Next to drinking cultures and the extent of stigmatization of AUDs, the treatment systems vary across Europe [44, 45, 9]. Thereby treatment barriers should be investigated in the context of the specific health care system.

The wish not to stop drinking completely was particularly frequent among the treatment barriers reported in this study. Treatment approaches that emphasize goals other than abstinence [46, 47] could be reconsidered for those patients. For example, ‘guided self-change’ (GSC) is a brief cognitive-behavioral motivational intervention designed to assist in recognizing and resolving alcohol-related problems [48]. It includes goal setting, self-monitoring of drinking behavior, analysis of drinking situations, and learning alternate coping skills [49].

### Strengths and weaknesses

This study was one of the largest undertaken to investigate reasons for not seeking treatment on representative samples of primary care patients, with a good refusal rate of below 20 % on the individual level. We were able to investigate reasons for not seeking treatment on a sample that had not yet received treatment for AUDs and thereby varied with respect to AUDs severity and other patient characteristics that tend to be more homogenous among patients in specialized care [50]. Furthermore, this study combined information from open ended and closed questions thus including yet unknown or less investigated reasons. When we compared this study with the only study with a comparable sample size [3], the present study was able to also investigate 12 months answers which were found to be somehow different from lifetime answers and less prone to memory biases.

The main limitations of the present study were the cross-sectional design, neither allowing any causal interpretation nor clear assessment of temporal sequences as suggested by Saunders’ model. Although the GP’s refusal rate of 56.4 % was considerable, as other studies with register-based random sampling usually have lower response rates [51, 52] it seems overall acceptable. As reported above, the majority of GPs refused study participation for reasons unrelated to this study. However, some GPs reported doubts about releasing patient data despite approval from research ethics boards and assurance of full anonymity and very few GPs were afraid of losing patients given the nature of the study on patients’ alcohol use which is a stigmatized subject [9]. Therefore it cannot be excluded completely that those GPs who refused study participation have a different patient population than the participants of this study. Furthermore, due to the low response rate in the open ended questions we can only speculate about those who did not give any reasons. While we could not find any patient characteristics predictive of reporting a reason for not seeking treatment for lifetime AUDs, participants with a severe AUDs diagnosis were less likely to report a reason as compared to persons with a light AUDs diagnosis. It is possible that those patients with severe AUDs did not report a reason because they did not acknowledge the problem or perceived a higher stigmatization. Overall, as reflected in the decreased likelihood to report any reason for not seeking treatment as well as in the discontinuation of observed trends in the reported reasons, this group of patients seems to be a special group and the respective results should be interpreted with caution.

## Conclusion

General health care patients from six European countries were asked for reasons why they did not seek help for AUDs. The study showed that lacking problem awareness was the major reason for not seeking treatment, especially in less severe cases that are however prone to physical harm from heavy drinking [36]. A more regular monitoring of patients’ alcohol consumption and offering brief interventions for individuals with mild AUDs could potentially improve their health and reduce societal harm [53]. For more severe cases treatment barriers were reported more frequently. For these cases GPs could serve as a junction point to specialized care.

## Additional file

Additional file 1: Table S1. Ethical review by country. **Table S2** Overview on coding for open ended and closed questions on treatment seeking for alcohol use disorders. **Table S3** Frequencies, weighted percentages including 95 % confidence intervals (CI) and standardized residuals (Res) for reasons not to seek treatment by country. Refers to patients that were diagnosed with an alcohol use disorder, reported a reason for not seeking treatment, and that did not receive treatment. Multiple reasons per patient were possible. (DOCX 25 kb)

## Abbreviations

AUD: Alcohol use disorder
GP: general practitioner
CIDI: Composite International Diagnostic Interview
DSM: Diagnostic and Statistical Manual of Mental Disorders
CI: Confidence interval
