# Supplementary material for: Alcohol use disorder severity and reported reasons not to seek treatment: a cross-sectional study in European primary care practices
Source: Subst Abuse Treat Prev Policy. 2015 Aug 12;10:32. doi: 10.1186/s13011-015-0028-z (PMC4534056; doi:10.1186/s13011-015-0028-z)
Supplement: Additional file 1: Table S1. — Ethical review by country. Table S2 Overview on coding for open ended and closed questions on treatment seeking for alcohol use disorders. Table S3 Frequencies, weighted percentages including 95 % confidence intervals (CI) and standardized residuals (Res) for reasons not to seek treatment by country. Refers to patients that were diagnosed with an alcohol use disorder, reported a reason for not seeking treatment, and that did not receive treatment. Multiple reasons per patient were possible. (DOCX 25 kb) [file 13011_2015_28_MOESM1_ESM.docx]

Table A1 Ethical review by country.

| **Country** | **Institution** | **Obtained ethics approval on:** | **Reference number** |
| --- | --- | --- | --- |
| Germany | TU Dresden | August 28, 2012 | EK 207072012 |
| Hungary | Corvinus University Budapest | June 20, 2013 | N/A |
| Italy1 | Servizio Sanitario Nazionale | May 16, 2013 | 8/13 |
| Italy2 | Agenzia Regionale di Sanità Toscana | May 2, 2013 | 14580 |
| Latvia | Riga East Clinical University Hospital | March 7, 2013 | 01-20/1102 |
| Poland | Medical University Warsaw | August 21, 2012 | KB/205/2012 |
| Spain | Hospital Clinic Barcelona | July 27, 2012 | 2012/7770 |

Table A2 Overview on coding for open ended and closed questions on treatment seeking for alcohol use disorders.

| Variable/broader category | Subcategories  (12 month and lifetime) | Closed question (yes/no)  (12 month) |
| --- | --- | --- |
| Lack of awareness | Patient did not see any problem |  |
|  | Patient stated that no help was needed |  |
|  | Consumption was considered as normal or adequate |  |
|  | Drinking behavior was attributed to youth |  |
|  | Drinking was considered as under control |  |
|  | Drinking was not considered as serious |  |
| Cope alone | Intention to cope with problem alone |  |
|  | Patient reduced or stopped drinking |  |
|  | Patient received some kind of non-professional support |  |
|  | Acknowledgement of the problem to a certain degree, but not of its severity |  |
| Shame or stigma | Fear of stigma and shame | Were you too ashamed to ask for help?  Did you fear being stigmatized for alcohol problems? |
|  | Social pressure |  |
|  | Patient considered his/her drinking as a private issue |  |
| Other problem | Some other problem was the reason for his/her drinking |  |
|  | Consumption of other substance was more prominent |  |
| Encounter barriers | Lack of possibility or knowledge |  |
|  | Lack of time |  |
|  | Patient asked for help but did not get any |  |
|  | Treatment was not affordable |  |
|  | Wish not to stop drinking | Did you fear that you would be told to stop drinking completely? |
|  | Patient did not consider treatment as an option |  |
|  | No trust in treatment system or effectiveness |  |
|  |  | Did the help system not offer the kind of help you were seeking? |

Table A3 Frequencies, weighted percentages including 95% confidence intervals (CI) and standardized residuals (Res) for reasons not to seek treatment by country. Refers to patients that were diagnosed with an alcohol use disorder, reported a reason for not seeking treatment, and that did not receive treatment. Multiple reasons per patient were possible.

|  | Latvia | | | | Spain | | | | Germany | | | | Hungary | | | | Italy | | | | Poland | | | |
| --- | --- | --- | --- | --- | --- | --- | --- | --- | --- | --- | --- | --- | --- | --- | --- | --- | --- | --- | --- | --- | --- | --- | --- | --- |
|  | N | % | 95% CI | Res | N | % | 95% CI | Res | N | % | 95% CI | Res | N | % | 95% CI | Res | N | % | 95% CI | Res | N | % | 95% CI | Res |
| Lack of problem awareness | 41 | 47.8 | 37.1-58.6 | -0.9 | 0 | 0 | - | -3.6 | 59 | 87.2 | 78.8-95.6 | 3.7 | 14 | 42.4 | 25.5-59.4 | -0.4 | 25 | 59.2 | 43.0-75.4 | -0.1 | 0 | 0 | - | -1.1 |
| Cope alone | 36 | 41.7 | 31.1-52.3 | 4.2 | 0 | 0 | - | -2.2 | 8 | 12.8 | 4.4-21.2 | -1.6 | 4 | 12.1 | 0.9-23.3 | -0.8 | 3 | 5.7 | 0.0-12.0 | -2.2 | 2 | 100 | - | 2.2 |
| Shame or stigma | 16 | 18.3 | 10.1-26.6 | -1.8 | 15 | 71.4 | 52.0-90.9 | 3.2 | 9 | 13.6 | 5.2-21.9 | -2.3 | 16 | 48.5 | 31.3-65.6 | 2.7 | 17 | 39.2 | 23.2-55.2 | 1.0 | 0 | 0 | - | -0.8 |
| Other problem | 0 | 0 | - | -1.0 | 0 | 0 | - | -0.5 | 3 | 4.3 | 0.0-9.2 | 2.5 | 0 | 0 | - | -0.6 | 0 | 0 | - | -0.7 | 0 | 0 | - | -0.2 |
| Encounter barriers | 19 | 21.5 | 12.8-30.1 | -0.3 | 15 | 71.4 | 52.0-90.9 | 4.1 | 7 | 10.6 | 3.1-18.2 | -2.2 | 3 | 9.1 | 0.0-19.0 | -1.4 | 15 | 32.8 | 18.0-47.5 | 1.2 | 1 | 54.5 | 0.0-100.0 | 0.6 |
